# Supplementary material for: Abscisic acid positively regulates rice spikelet closure
Source: PLoS One. 2026 May 20;21(5):e0349343. doi: 10.1371/journal.pone.0349343 (PMC13189316; doi:10.1371/journal.pone.0349343)
Supplement: S1 Fig — (A) Xingan Zaozhan, (B) Jiazao 70, (C) Zhenshan 97B. (DOC) [file pone.0349343.s001.doc]

A


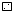
0mg/L
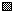
50mg/L
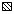
100mg/L
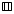
200mg/L
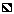
400mg/L
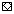
800mg/L(Xingan Zaozhan)

B


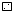
0mg/L
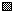
50mg/L
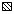
100mg/L
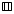
200mg/L
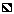
400mg/L
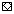
800mg/L(Jiazao 70)

C


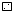
0mg/L
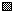
50mg/L
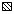
100mg/L
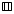
200mg/L
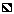
400mg/L
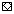
800mg/L (Zhenshan 97*B*)

Figure 1. Effect of ABA on spikelet closure in three fertile rice varieties. (Figure 1A) Xingan Zaozhan, (Figure 1B) Jiazao 70, (Figure 1C) Zhenshan 97B. L：left figure，R：right figure.

Lowercase letters a, b, c... indicate significant differences, while Capital letters A, B, C... indicate highly significant differences.The concentrations of ABA at 50, 100, 200, 400, and 800 mg/L correspond to 0.19, 0.38, 0.76, 1.51, and 3.03 mM, respectively. The data in this figure are the means and standard deviations of three independent samples.
