# Supplementary material for: Abscisic acid positively regulates rice spikelet closure
Source: PLoS One. 2026 May 20;21(5):e0349343. doi: 10.1371/journal.pone.0349343 (PMC13189316; doi:10.1371/journal.pone.0349343)
Supplement: S2 Fig — (A) Qiyuan S, (B) Yue 4A, (C) Zhenshan 97A. (DOC) [file pone.0349343.s002.doc]

A


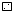
0mg/L
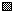
50mg/L
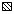
100mg/L
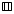
200mg/L
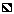
400mg/L
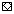
800mg/L (Qiyuan S)

B


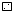
0mg/L
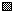
50mg/L
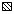
100mg/L
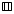
200mg/L
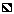
400mg/L
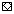
800mg/L (Yue 4A)

C


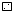
0mg/L
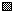
50mg/L
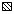
100mg/L
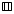
200mg/L
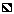
400mg/L
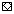
800mg/L(Zhenshan 97A)

Figure 2. Effect of ABA on spikelet closure in three sterile rice varieties. (Figure 2A) Qiyuan S, (Figure 2B) Yue 4A, (Figure 2C) Zhenshan 97A.L：left figure，R：right figure.Lowercase letters a, b, c... indicate significant differences, while Capital letters A, B, C... indicate highly significant differences.The concentrations of ABA at 50, 100, 200, 400 and 800 mg/L correspond to 0.19, 0.38, 0.76, 1.51 and 3.03 mM, respectively. The data in this figure are the means and standard deviations of three independent samples.
