# Supplementary material for: Abscisic acid positively regulates rice spikelet closure
Source: PLoS One. 2026 May 20;21(5):e0349343. doi: 10.1371/journal.pone.0349343 (PMC13189316; doi:10.1371/journal.pone.0349343)
Supplement: S3 Fig — (A) Xingan Zaozhan, (B) Jiazao 70, (C) Zhenshan 97B. (DOC) [file pone.0349343.s003.doc]

A


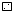
0mg/L
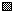
5mg/L
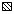
10mg/L
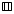
20mg/L
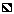
40mg/L
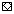
80mg/L (Xingan Zaozhan)

B


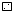
0mg/L
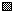
5mg/L
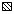
10mg/L
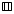
20mg/L
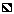
40mg/L
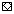
80mg/L (Jiazao 70)

C


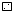
0mg/L
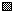
5mg/L
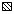
10mg/L
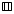
20mg/L
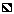
40mg/L
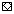
80mg/L (Zhenshan 97B)

Figure 3. Effect of FL on spikelet closure in three fertile rice varieties. (Figure 3A) Xingan Zaozhan, (Figure 3B) Jiazao 70, (Figure 3C) Zhenshan 97B.L：left figure，R：right figure.Lowercase letters a, b, c... indicate significant differences, while Capital letters A, B, C... indicate highly significant differences.The concentrations of FL at 5, 10, 20, 40, and 80 mg/L correspond to 0.015, 0.030, 0.061, 0.121, and 0.243 mM, respectively. The data in this figure are the means and standard deviations of three independent samples.
