# Supplementary material for: Abscisic acid positively regulates rice spikelet closure
Source: PLoS One. 2026 May 20;21(5):e0349343. doi: 10.1371/journal.pone.0349343 (PMC13189316; doi:10.1371/journal.pone.0349343)
Supplement: S4 Fig — (A) Qiyuan S, (B) Yue 4A, (C) Zhenshan 97A. (DOC) [file pone.0349343.s004.doc]

A


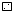
0mg/L
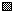
5mg/L
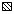
10mg/L
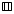
20mg/L
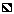
40mg/L
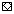
80mg/L (Qiyuan S)

B


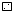
0mg/L
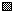
5mg/L
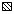
10mg/L
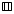
20mg/L
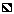
40mg/L
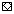
80mg/L (Yue 4A)

C


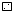
0mg/L
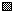
5mg/L
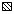
10mg/L
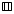
20mg/L
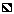
40mg/L
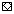
80mg/L (Zhenshan 97A)

Figure 4. Effect of FL on spikelet closure in three sterile rice varieties. (Figure 4A) Qiyuan S, (Figure 4B) Yue 4A, (Figure 4C) Zhenshan 97A.L：left figure，R：right figure. Lowercase letters a, b, c... indicate significant differences, while Capital letters A, B, C... indicate highly significant differences.The concentrations of FL at 5, 10, 20, 40, and 80 mg/L correspond to 0.015, 0.030, 0.061, 0.121 and 0.243 mM, respectively. The data in this figure are the means and standard deviations of three independent samples.
