# Supplementary material for: Abscisic acid positively regulates rice spikelet closure
Source: PLoS One. 2026 May 20;21(5):e0349343. doi: 10.1371/journal.pone.0349343 (PMC13189316; doi:10.1371/journal.pone.0349343)
Supplement: S5 Fig — (DOC) [file pone.0349343.s005.doc]

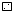
the opening palea and lemma
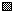
 palea and lemma with max-angle opening,
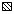
the closed palea and lemma

Figure 5. Endogenous levels of ABA in rice lodicules. L：left figure，R：right figure.(L: Xinganzaozhan; R: Qiyuan S).Lowercase letters a, b, c... indicate significant differences, while Capital letters A, B, C... indicate highly significant differences.
