# Supplementary material for: Abscisic acid positively regulates rice spikelet closure
Source: PLoS One. 2026 May 20;21(5):e0349343. doi: 10.1371/journal.pone.0349343 (PMC13189316; doi:10.1371/journal.pone.0349343)
Supplement: S6 Fig — (DOC) [file pone.0349343.s006.doc]

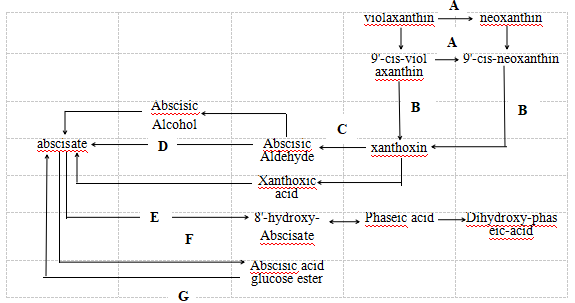


Figure 6. ABA biosynthesis pathway. (A) Neoxanthin synthase; (B) 9-cis-epoxycarotenoid dioxygenase (NCED); (C) Xanthoxin dehydrogenase; (D) Abscisic aldehyde oxidase; (E) (+)-Abscisic acid 8'-hydroxylase; (F) Abscisate β-glucosyltransferase; (G) β-D-glucopyranosyl abscisate β-glucosidase.
