# Supplementary material for: Abscisic acid positively regulates rice spikelet closure
Source: PLoS One. 2026 May 20;21(5):e0349343. doi: 10.1371/journal.pone.0349343 (PMC13189316; doi:10.1371/journal.pone.0349343)
Supplement: S7 Fig — (DOC) [file pone.0349343.s007.doc]

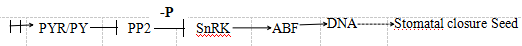


Figure 7. ABA signal transduction.PYR/PYL: ABA receptor PYR/PYL family; PP2C: protein phosphatase 2C; SnRK2: serine/threonine-protein kinase SRK2; ABF: ABA-responsive element binding factor.
