# Supplementary figures and images for: Abscisic acid positively regulates rice spikelet closure
Source: PLoS One. 2026 May 20;21(5):e0349343. doi: 10.1371/journal.pone.0349343 (PMC13189316; doi:10.1371/journal.pone.0349343)

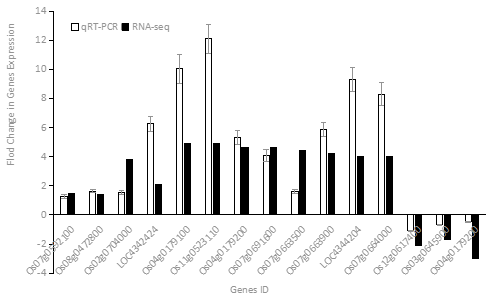


Figure 8. Comparation of the differentially expressed genes between qRT-PCR and RNA-seq

Supplement: S8 Fig — (DOC) [file pone.0349343.s008.doc]
