# Supplementary material for: Abscisic acid positively regulates rice spikelet closure
Source: PLoS One. 2026 May 20;21(5):e0349343. doi: 10.1371/journal.pone.0349343 (PMC13189316; doi:10.1371/journal.pone.0349343)
Supplement: S9 Fig — (DOC) [file pone.0349343.s009.doc]

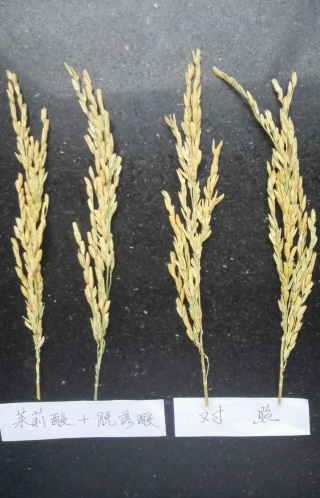

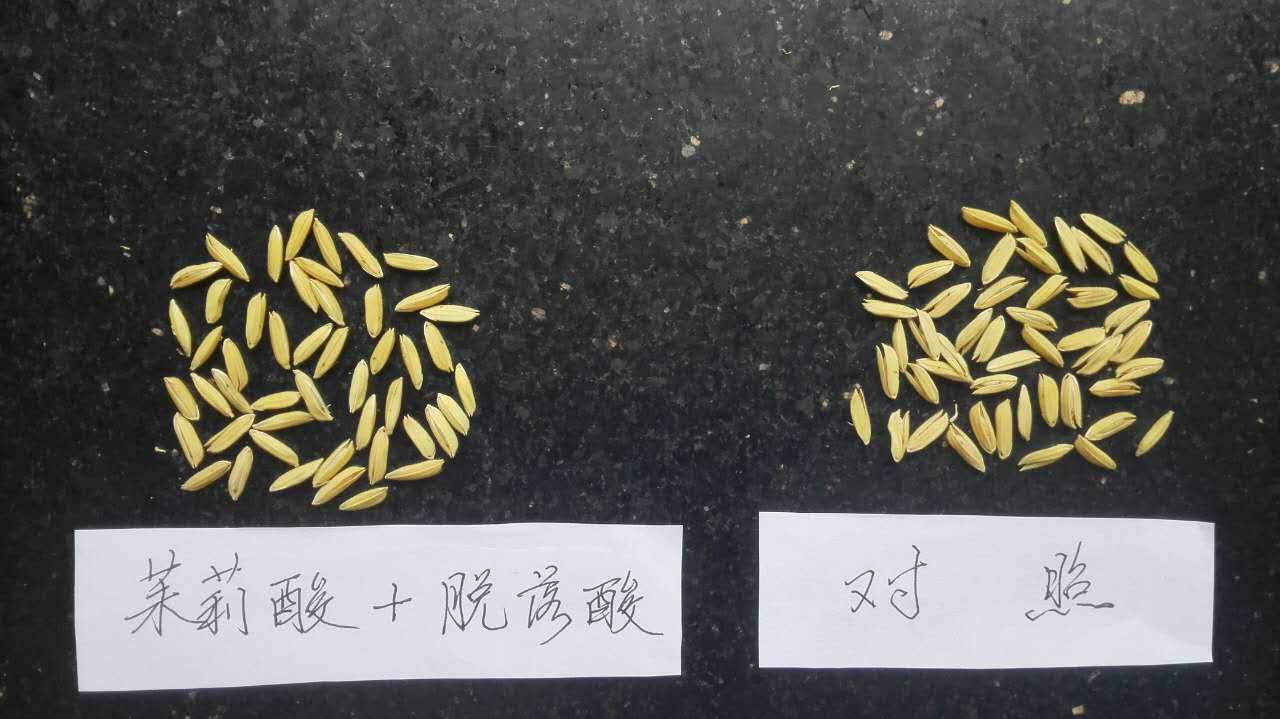


MeJA+ABA

CK

MeJA+ABA

CK

Figure 9. Influence of (MeJA+ABA) on female parent spikelet closure (Qiyuan S/Xingan Zaozhan).
