# Supplementary material for: Abscisic acid positively regulates rice spikelet closure
Source: PLoS One. 2026 May 20;21(5):e0349343. doi: 10.1371/journal.pone.0349343 (PMC13189316; doi:10.1371/journal.pone.0349343)
Supplement: Attachment — Submitted filename: 3.Response to Reviewers.doc [file pone.0349343.s012.doc]

Dear Editors and Reviewers,

I am Youming Huang,the submitting author of the manuscript "Abscisic acid positively regulates rice spikelet closure" (PONE-D-25-60666).Many thanks to the editor and the reviewers for their dedication to my paper.All authors of this manuscript have made careful and thorough revisions，and have provided responses to every query from the editor and reviewers. I will now present our point-by-point responses:

To editors:
1. Please ensure that your manuscript meets PLOS ONE's style requirements, including those for file naming. The PLOS ONE style templates can be found at <https://journals.plos.org/plosone/s/file?id=wjVg/PLOSOne_formatting_sample_main_body.pdf> and <https://journals.plos.org/plosone/s/file?id=ba62/PLOSOne_formatting_sample_title_authors_affiliations.pdf>

**Respond：**We have compared our manuscript against PLOS ONE's style requirements one by one, including font, line spacing, file naming, and references. It now meets PLOS ONE's style requirements.

2. In your Methods section, please provide additional information regarding the permits you obtained for the work. Please ensure you have included the full name of the authority that approved the field site access and, if no permits were required, a brief statement explaining why.

**Respond：**No permits were required, as this study investigated the physiological mechanisms of rice (Oryza sativa ).The study does not pertain to ethical issues, environmental protection, or wildlife collection, and no personal conflict of interest exists.

3. Thank you for stating the following in the Acknowledgments Section of your manuscript: “National Natural Science Foundation Project (31360297)”We note that you have provided funding information that is not currently declared in your Funding Statement. However, funding information should not appear in the Acknowledgments section or other areas of your manuscript. We will only publish funding information present in the Funding Statement section of the online submission form. Please remove any funding-related text from the manuscript and let us know how you would like to update your Funding Statement. Currently, your Funding Statement reads as follows: “Xiaochun Zeng（XCZ）the National Natural Science Foundation Project (31360297) National Natural Science Foundation of China https://www.nsfc.gov.cn/”Please include your amended statements within your cover letter; we will change the online submission form on your behalf.

**Respond：**"'National Natural Science Foundation Project (31360297)' has been deleted from the manuscript.

Please revise the funding statement to“**Grant (No:31360297) from the National Natural Science Foundation of China to Xiaochun Zeng**”，and kindly add this grant to the online submission form's funding section for me. Thank you!"

4. We note that the grant information you provided in the ‘Funding Information’ and ‘Financial Disclosure’ sections do not match. 
When you resubmit, please ensure that you provide the correct grant numbers for the awards you received for your study in the ‘Funding Information’ section.

**Respond：**The grant information provided in the 'Funding Information' and 'Financial Disclosure' sections is consistent for this study.

“Grant (No:31360297) from the National Natural Science Foundation of China to Xiaochun Zeng”.Please kindly add this grant to the online submission form's ‘Funding Information’ for me. Thank you!"

5. Thank you for stating the following financial disclosure: “Xiaochun Zeng（XCZ）the National Natural Science Foundation Project (31360297) National Natural Science Foundation of China [https://www.nsfc.gov.cn/”](https://www.nsfc.gov.cn/\)

Please state what role the funders took in the study.  If the funders had no role, please state: "The funders had no role in study design, data collection and analysis, decision to publish, or preparation of the manuscript." If this statement is not correct you must amend it as needed. Please include this amended Role of Funder statement in your cover letter; we will change the online submission form on your behalf.

**Respond：**The funders had no role in study design, data collection and analysis, decision to publish, or preparation of the manuscript.

6. We note that your Data Availability Statement is currently as follows: “All relevant data are within the manuscript and its Supporting Information files.”Please confirm at this time whether or not your submission contains all raw data required to replicate the results of your study. Authors must share the “minimal data set” for their submission. PLOS defines the minimal data set to consist of the data required to replicate all study findings reported in the article, as well as related metadata and methods (https://journals.plos.org/plosone/s/data-availability#loc-minimal-data-set-definition).

For example, authors should submit the following data:

- The values behind the means, standard deviations and other measures reported;
- The values used to build graphs;
- The points extracted from images for analysis.

Authors do not need to submit their entire data set if only a portion of the data was used in the reported study.

If your submission does not contain these data, please either upload them as Supporting Information files or deposit them to a stable, public repository and provide us with the relevant URLs, DOIs, or accession numbers. For a list of recommended repositories, please see <https://journals.plos.org/plosone/s/recommended-repositories.>

If there are ethical or legal restrictions on sharing a de-identified data set, please explain them in detail (e.g., data contain potentially sensitive information, data are owned by a third-party organization, etc.) and who has imposed them (e.g., an ethics committee). Please also provide contact information for a data access committee, ethics committee, or other institutional body to which data requests may be sent. If data are owned by a third party, please indicate how others may request data access.

**Respond：**

（1）We hereby certify that the submitted manuscript includes the complete raw data set necessary to reproduce the recults reported in our study.
（2）The “minimal data set ”required to replicate the findings is available in the attached file.

（3）The RNA-seq raw data of this study has not yet been fully analyzed and processed; it remains in the confidential stage and therefore has not been deposited in a public repository. We appreciate your understanding.

7. PLOS requires an ORCID iD for the corresponding author in Editorial Manager on papers submitted after December 6th, 2016. Please ensure that you have an ORCID iD and that it is validated in Editorial Manager. To do this, go to ‘Update my Information’ (in the upper left-hand corner of the main menu), and click on the Fetch/Validate link next to the ORCID field. This will take you to the ORCID site and allow you to create a new iD or authenticate a pre-existing iD in Editorial Manager.

**Respond：Already validated.**

8. If the reviewer comments include a recommendation to cite specific previously published works, please review and evaluate these publications to determine whether they are relevant and should be cited. There is no requirement to cite these works unless the editor has indicated otherwise. 
**Respond：**The referee's remarks contain no recommendations regarding the citation of particular prior publications.

**To reviewer #1:**

Reviewer #1: Overall Recommendation:Major Revision
General Comments:
This study investigates the role of abscisic acid (ABA) in regulating rice spikelet closure using physiological, molecular, and field application approaches. The topic is of significant agronomic importance, especially for hybrid rice seed production. The experimental design is generally sound, and the combination of hormone treatments, RNA-seq, and field trials provides a comprehensive approach. However, several issues need to be addressed before the manuscript can be considered for publication.

Major Comments:
1.Lack of Statistical Details:
While the manuscript mentions statistical tests (e.g., F-tests, multiple comparisons), it does not provide sufficient details on the statistical methods used, such as the specific type of ANOVA, post-hoc tests, or sample sizes for each experiment. Please include these details in the Methods section and ensure that all figures include clear indications of statistical significance (e.g., asterisks with defined p-values).
**Respond：**

We have made substantial revisions to the details of the statistical methods：

Data analysis was similar to what we used previously. The data in the Figs and tables

were the means and standard deviations of three biological sets of data with each biological set

came from three technical measurements. Mean values within table columns were analyzed

with Duncan new multiple range test. Values in a column with different letters indicate a

significant different difference among the floret stages. Uppercase and lowercase letters repre-

sent significant differences between floret stages at ρ = 0.01 and ρ = 0.05, respectively.
**（See manuscript）**
2.RNA-Seq Data Availability:
The manuscript states that "all relevant data are within the manuscript and its Supporting Information files." However, RNA-seq data are not typically included in the main text or supplementary files. The raw or processed RNA-seq data should be deposited in a public repository (e.g., NCBI GEO or SRA), and the accession number must be provided.
**Respond：**We have not deposited the raw or processed RNA-seq data in a public repository.As the RNA-seq raw data of this study have not yet been fully analyzed and processed, they remain in the confidential stage. We appreciate your understanding.

3.Hormonal Interactions:
The study focuses on ABA but also involves MeJA. However, the interaction between ABA and other hormones (e.g., IAA, GA) is only briefly discussed. Given the complexity of hormonal regulation, more discussion or data on how ABA interacts with other hormones during spikelet closure would strengthen the manuscript.
**Respond：**Given the complexity of hormonal regulation, we have made the following revisions to strengthen the manuscript:

MeJA is currently recognized as the most potent inducer of floret opening, effective in various graminaceous plants including rice, wheat, and sorghum^[16]^. It promotes floret opening by enhancing ATPase and CAT activities in rice lodicules, facilitating the accumulation of osmotic regulatory substances (such as soluble sugars and ascorbic acid), reducing H2O₂concentration, enabling lodicule water absorption and swelling, and ultimately driving floret opening^[9]^. The present study demonstrates that exogenous ABA treatment significantly increases the floret closing rate, shortens floret opening duration, and strongly promotes floret closure. Although MeJA and ABA possess independent biosynthetic pathways representing a metabolic parallel relationship, cross-talk exists between them: ABA → SAPK10 (SnRK2 family kinase) → bZIP72 (transcription factor) → AOC gene expression → increased JA/MeJA synthesis. Upon autophosphorylation, SAPK10 phosphorylates bZIP72, enhancing its protein stability and DNA-binding capacity, thereby promoting AOC promoter activity^[53]^. Future research should further elucidate the spatiotemporal specificity mechanisms underlying ABA-JA metabolic cross-regulation during floret opening, particularly the activity status of the SAPK10-bZIP72-AOC module in lodicule cells.

**（See manuscript）**

[53]Cui Huan, Gao Qiaoli, Luo Lixin, Yang Jing, Chen Chun, Guo Tao, Liu Yongzhu,Huang Yongxiang, Wang Hui, Chen Zhiqiang, Xiao Wuming.Transcriptome Analysis of Hormone Signal Transduction and Glutathione MetabolicPathway in Rice Seeds at Germination Stage[J].Chinese Journal of Science, 2021, 35(6): 554－564.

4.Mechanistic Insight:
While RNA-seq identified DEGs related to ABA synthesis and signaling, functional validation (e.g., mutant analysis, transgenic plants) is lacking. The authors should acknowledge this limitation and discuss how future studies could address it.
**Respond：**We have added this discussion in the revised manuscript.

Yes, through RNA-seq sequencing, we identified a large number of differentially expressed genes (DEGs) related to ABA biosynthesis and degradation as well as ABA signal transduction, such as xanthoxin dehydrogenase genes, phosphatase genes in the PP2C family, and so on. In this study, we did not validate their functions (e.g., mutant analysis, transgenic plants), which is a limitation of our work. In future research, we will conduct in-depth studies by combining proteomics.

5.Field Application Data:
The field trial results are promising, but the sample size and replication are not clearly described. Please specify the number of biological and technical replicates for the field experiments and whether the trials were repeated across multiple seasons or locations.
**Respond：**Thank you for your thorough and careful review.

This has been modified to clarify the sample size and reproducibility**（See manuscript）.**

The experiment was not conducted across multiple seasons or locations, because the aim of this study was to explore the mechanism of ABA-mediated rice floret closure. In the future, we will increase replication across multiple seasons and locations to prepare for the practical application of ABA and MeJA in hybrid rice seed production.

Minor Comments:
Language and Clarity:
The manuscript would benefit from professional English editing to improve clarity and flow. Some sentences are overly long or awkwardly phrased.
**Respond：**We have carefully and thoroughly polished the entire manuscript, including word choice, sentence structure, grammar, and sentence length, to better conform to the conventions of scientific writing.
（See manuscript）

Figure and Table Quality:
Some figures (e.g., Fig. 1–4) are difficult to interpret due to overlapping labels and unclear legends. Please improve the resolution and organization of figures.
Table 3 (primers) is in Chinese. All content should be in English for international readability.
**Respond：**

（1）Regarding the issue of overlapping labels and unclear legends, we have improved the resolution of the figures and enhanced their organization.

（2）The Chinese phrases have been translated into English. (See manuscript:序号--No. 基因ID--Gene ID 正向引物--Forward primer 反向引物--Reverse primer)

Introduction and Discussion:
The introduction could better contextualize the study within the broader literature on ABA and spikelet dynamics.
**Respond：** The introduction has been revised to better contextualized the study within the literature on ABA and spikelet dynamics.

To verify the effect of ABA in promoting rice spikelet closure, and more importantly, to explore its mechanism and identify additional regulatory techniques for rice spikelet closure, this study tested the effects of ABA and the ABA biosynthesis inhibitor FL on rice spikelet closure using three fertile and three sterile rice lines, monitored the dynamic levels of endogenous ABA in lodicules during the closure process, and further analyzed the differential expression of key enzyme genes involved in ABA biosynthesis and degradation and genes related to ABA signal transduction in lodicules at the transcriptional level during spikelet closure.

（See manuscript）

The discussion should more directly link the molecular findings (e.g., up-regulation of PYL4, down-regulation of PP2C) to the physiological observations.

**Respond：We have added the following content in the discussion:**

During the critical transition period from floret opening to closing, the β-carotene hydroxylase, the FAD-dependent urate hydroxylase, ZEP, NCED, and the xanthinaldehyde dehydrogenase genes were all up-regulated, indicating elevated endogenous ABA levels in the floret, which is consistent with our measured results (Figure 5). Concurrently,the ABA receptor PYL4 was up-regulated, whereas three PP2C family phosphatases—PP2C66, PP2C8, and PP2C30 were down-regulated,and the SAPK6 from the SnRK2 family was up-regulated(Figure 7, Table 2). This expression pattern is consistent with the dual negative regulatory mechanism of ABA signaling,and also consistent with the ABA-promoted closure of opening florets observed in this study.Although RNA-seq sequencing identified a large number of DEGs related to ABA biosynthesis and degradation as well as ABA signal transduction, such as xanthoxin dehydrogenase genes and phosphatase genes in the PP2C family, regrettably, this study did not validate their functions (e.g., mutant analysis, transgenic plants). In future research, we will conduct in-depth studies by integrating proteomics.

（See manuscript）

Reference Formatting:
Some references are incomplete or inconsistently formatted (e.g., missing titles, journal names not abbreviated). Please ensure all references follow PLOS ONE guidelines.
**Respond：**Every reference has been carefully checked and has been revised and supplemented.（See the reference）

Conclusion:This manuscript presents valuable insights into the role of ABA in rice spikelet closure and has potential applications in hybrid seed production. With revisions addressing the above points, it would be suitable for publication in PLOS ONE.

**To reviewer #2:**

Reviewer #2: The manuscript "Abscisic acid positively regulates rice spikelet closure " is a well thought research reaching to a conclusion that reflects solution to the concern raised on spikelet closure. However, there are several issues that must be taken care:
1.I struggled to find in the introduction section of the manuscript why the authors chose to use combination of ABA and MeJA used for spraying. Ultimately I found the answer in the methodology section (L197), which in fact should have been stated in the introduction section.
**Respond：**I sincerely appreciate the reviewer's conscientious and responsible attitude, and deeply admire your dedication to the work.The content has been moved to the introduction section, with revisions and additions made accordingly：

Xiaochun Zeng et al. found that Jasmonic acid (JA) and Methy Jasmonate (MeJA) can promote the opening of mature rice spikelets^[16]^，and the effect is more......

（See manuscript）

2.if both Jasmonic acid (JA) and Methyl Jasmonate (MeJA) can promote the opening of mature rice spikelets, why only MeJA has been considered for the study?

**Respond：**MeJA can enter plants through stomata and be hydrolyzed to JA by esterases in the cytoplasm; MeJA is far less expensive than JA; MeJA is readily soluble in organic solvents such as ethanol, methanol, and DMSO, but almost insoluble in water, whereas JA is only slightly soluble in organic solvents and highly volatile; most importantly, MeJA is far more effective than JA in inducing rice floret opening.

3.The authors have considered 3 fertile and 3 rice varieties were considered, but the results obtained for them have not been discussed with regard to any difference the closure of stomata among them. Thus the reason of considering 3 rice varieties in both the sterile and fertile group remained unaddressed.
**Respond：**The **f**ertile rice florets basically close without any obstacles , while most sterile lines have obstacles in floret closing—some do not close tightly, and some split completely, resulting in split-spikelet seeds that seriously affect the quality of hybrid seeds. This study selected three fertile and three sterile rice varieties (among the sterile varieties, there were both two-line and three-line types, both indica and japonica types) as experimental materials, aiming to understand the universality of ABA's effect in promoting rice floret closing.Despite variations among varieties, the overall response pattern was consistent.

（See the discussion）

4.All the figures in their right panel need to state in the caption the 'alphabets' used against the top of each column.
**Respond：**The following information has been added to the figure legend:Lowercase letters a, b, c... indicate significant differences, while Capital letters A, B, C... indicate highly significant differences.

Modified（See fig.1--fig.5）

5. The discussion section is without citation of any figures. Besides, the discussion section does not seem to interpret the results depicted in all the figures. It appears the discussion is independent of the results.

**Respond：The discussion section in the revised manuscript has been better linked to the results.**

**for example**

(1) Figure 1-Figure 4 indicated,despite variations among varieties, the overall response pattern was consistent.

(2)To clarify this effect, we employed FL(Figure 3 and Figure 4), which...

(3)However, this study solely examined endogenous ABA levels(Figure 5) and lacked insight into...

(4)Although MeJA and ABA possess independent biosynthetic pathways representing a metabolic parallel relationship(Figure 6),

and so on **（See** the discussion**）**

**To reviewer #3:**

Reviewer #3: This manuscript addresses the role of abscisic acid (ABA) in regulating rice spikelet closure and explores its potential application in hybrid rice seed production. To address this question, the authors applied exogenous ABA and the ABA biosynthesis inhibitor fluoridone, analyzed transcriptome changes in lodicules, examined the agronomic effects of combined ABA and methyl jasmonate treatment. The transcriptome analysis suggests coordinated regulation of ABA biosynthesis and signaling genes during spikelet movement, and the combined hormone treatment is reported to enhance spikelet closure and hybrid seed yield.

While the topic is agronomically relevant, and the authors have generated a substantial amount of physiological, hormonal, and transcriptomic data, the manuscript in its current form contains fundamental issues in structure, rigor, and presentation. The biological question is not clearly framed, the Results section lacks synthesis and clarity, and key methodological and statistical details are insufficiently described. Addressing these problems would require a comprehensive reworking of the manuscript rather than a standard revision. I therefore recommend rejection, with the suggestion that the authors substantially revise and reorganize the manuscript before considering resubmission to an appropriate journal.

**Respond：**We have undertaken extensive, comprehensive, and thorough modifications to the manuscript, addressing its structure, academic rigor, and language expression.Thus rendering the biological problem framework more explicit, the results more synthesized and lucid, and the exposition of research methods and statistical particulars more exhaustive.

**Modified（See manuscript）**
